# A Bibliometric Analysis of 100 Most-Cited Articles on Corneal Cross-Linking

**DOI:** 10.3389/fmed.2022.904077

**Published:** 2022-06-01

**Authors:** Kaili Yang, Liyan Xu, Shaopei Wang, Meng Zhu, Qi Fan, Yuwei Gu, Yawen Wang, Qing Wang, Dongqing Zhao, Chenjiu Pang, Shengwei Ren

**Affiliations:** Henan Provincial People’s Hospital, Henan Eye Hospital, Henan Eye Institute, People’s Hospital of Zhengzhou University, Zhengzhou, China

**Keywords:** corneal cross-linking, 100 most-cited, bibliometric analysis, citation, VOSviewer

## Abstract

**Background:**

Highly cited papers are expected to have high-quality data that significantly contribute to the body of knowledge. The study aimed to evaluate the characters of the 100 most-cited articles on corneal cross-linking (CXL) through a bibliometric analysis.

**Materials and Methods:**

The Web of Science database was searched to identify papers published from 1950 to 2020. A bibliometric analysis of the top 100-cited articles was conducted in the current study. The citation differences between basic research, clinical research, and reviews were compared by Kruskal–Wallis test. The association between citations and publication year was evaluated by Spearman correlation analysis. The VOSviewer software was used to create networks of co-authorship and keywords map.

**Results:**

The median values of the number of citations, citations/year since publication, and citations since 2013 were 101, 9.5, and 11.92, respectively. A total of 61% of articles were clinical research. The citations since 2013 of clinical research were lower than basic research and the reviews (all *p* < 0.001). The publication year was positively correlated with the number of publications (*r* = 0.665, *p* = 0.013), and the total number of citations decreased for basic research (*r* = –0.447, *p* = 0.017), and clinical research (*r* = –0.433, *p* < 0.001). The *J REFRACT SURG* publishes the highest number of articles. The corresponding authors were predominantly from the Italy (*N* = 17), Germany (*N* = 16), and United States (*N* = 15). Spoerl Eberhard has the highest number of citations and total link strength with 15 articles. Extensive collaboration existed among the main core nodes containing “cross-linking (*N* = 45),” “riboflavin (*N* = 44),” and “ultraviolet A (UVA) (*N* = 42).”

**Conclusion:**

The present study focused on the comprehensive analysis of the top 100-cited articles on the CXL research, providing insight into research developments over the past decades.

## Introduction

The corneal cross-linking (CXL) is a surgery that could be achieved by utilizing the interaction between riboflavin and ultraviolet A (UVA) to create free radicals which then activate the normal physiological lysyl oxidase pathway (1, 2). CXL has become an effective and safe procedure when met specific criteria, and is used for the treatment of ectatic corneal diseases, especially keratoconus (3). In addition, several studies had reported the use of CXL in infectious keratitis, corneal edema, myopia, and other corneal diseases in clinical applications (4–6). Different CXL protocols had been proposed and different types of treatment had a progressive improvement over time (6, 7). The previous studies evaluated the efficacy and safety of different types of treatment, from classic treatment up to the accelerated treatment customized with regularization (8–11). With the improvement of surgical techniques and the increase of researcher numbers, the number of papers related to CXL has increased rapidly in recent years (6, 12). Thus, how to take an effectively quantitative method to better know the large number of articles is essential for researchers.

A bibliometric analysis encompasses the application of quantitative and statistical analyses of papers, which is a useful method to identify the total value of each paper (13). The bibliometric analysis can help researchers effectively to grasp the key information of a research field, and is widely used in a variety of research topics (14–16). As a relatively new ophthalmic surgery in the last two decades, the research on the bibliometric analysis on CXL researches was limited. A recent study of bibliometric analysis of CXL indicated that the number of publications gradually increased over time, especially since 2010 (17). It has been reported that the number of citations of a given paper is considered one of the measures of scientific merit, and highly cited papers are expected to have high-quality data that significantly contribute to the body of knowledge (18, 19). Thus, the current study aimed to conduct a bibliometric analysis to identify the 100 most frequently cited articles on CXL research from 1950 to 2020, and explore the citations, authors, journals, publishing countries, and keywords information of the top 100 papers.

## Materials and Methods

### Data Source and Research Process

The Institute for Scientific Information Web of Knowledge database from the Thomson Reuters Web of Science (WoS) Core Collection was used online as a data source for the current study. The topic was “corneal cross linking,” or “corneal cross-linking,” or “CXL” and the publication time ranged from 1950 to 2020. The article or review document type was included in the current analysis, and the retrieved results were saved as “Plain text” with “full record and cited references” (20). The most highly cited papers were carefully examined by authors, and the following criteria were used: paper focused on the CXL-related material science and surgical technique that did not specifically address CXL; paper introduced CXL that was only mentioned in the discussion and review that CXL is a small part.

Two authors (KY and LX) reviewed the data extraction process and verified any data problems due to human errors independently. If there were any discrepancies in evaluating the articles between the two authors, another author (SR) was asked to re-evaluate. One hundred articles with the highest citations on CXL research were included in the current analysis. If articles had an equal number of total citations, the more recent articles were ranked higher (15). The following information was collected from each article: title, publication date, journal name, the first author and the corresponding author, total number of citations, citations/year since publication, citations since 2013 (measured as the number of citations since 2013), research type (basic research, clinical research, or review), and keywords.

### Analytical Tool and Method

The citation data were presented as medians (P25, P75). The Kruskal–Wallis test was used, and other pairwise comparisons were performed to compare the citation differences between basic research, clinical research, and reviews. The Spearman correlation analysis was used to investigate the association between citation and publication year. The statistical analyses of the current study were performed using the SPSS 23, and a *p* < 0.05 (two-tailed) was considered to indicate statistical significance.

The VOSviewer software^1^ was used to create bibliometric networks of co-authorship and keywords map. The software assigns the nodes to clusters, with each cluster constituting a set of closely related nodes. Each cluster was represented by one color (21). More important terms had larger nodes, and strongly related terms were close to each other. The line between the nodes indicated a cooperative relationship, and a thicker line represented a stronger link between the two terms (14).

## Results

### Summary of 100 Articles

A total of 2,061 eligible publications related to CXL were searched in the current study. The 100 most-cited articles had a total of 14,844 citations (Supplementary Table 1). The publication year of the 100 most-cited articles was between 2003 and 2015. The total number of citations ranged from 68 to 1,619 (median: 101). The citations/year since publication ranged from 5.2 to 95.24 (median: 9.5), and the citations since 2013 ranged from 1 to 167 (median: 11.92).

### Citation Analysis According to the Article Type

In the current analysis, 28 articles were basic research, 61 articles were clinical research, and 11 articles were reviews (Table 1). The total numbers of citations for all articles, basic research, clinical research, and reviews were 27,855, 5,005, 17,408, and 5,442, respectively. The citations since 2013 of clinical research were lower than basic research and the reviews (all *p* < 0.001). No significant differences in the values of the total number of citations and citations/year since publication were found among the three article types (*p* > 0.05).

**TABLE 1:** Citations comparisons according to type of article, median (P25, P75).

Parameter	Total (*N* = 100)	Basic research (*N* = 28)	Clinical research (*N* = 61)	Review (*N* = 11)	P*	*P* ^#1^	*P* ^#2^	*P* ^#3^
Total citation	101.00 (81.00, 143.25)	117.50 (84.25, 165.00)	96.00 (77.50, 134.00)	117.00 (81.00, 138.00)	0.458	-	-	-
Citation/year since publication	11.92 (8.91, 15.92)	12.25 (9.94, 16.67)	11.67 (8.43, 15.46)	13.00 (9.20, 20.00)	0.761	-	-	-
Citation since 2013	11.00 (6.00, 118.75)	15.50 (11.00, 25.00)	8.00 (4.00, 12.50)	25.00 (15.00, 58.00)	<0.001	<0.001	0.639	<0.001

P*, Kruskal-Wallis Test; P^#1^, Basic research vs. Clinical research; P^#2^, Basic research vs. Review; P^#3^, Clinical research vs. Review.

### Annual Quantitative Distribution of Publications

**FIGURE 1:**
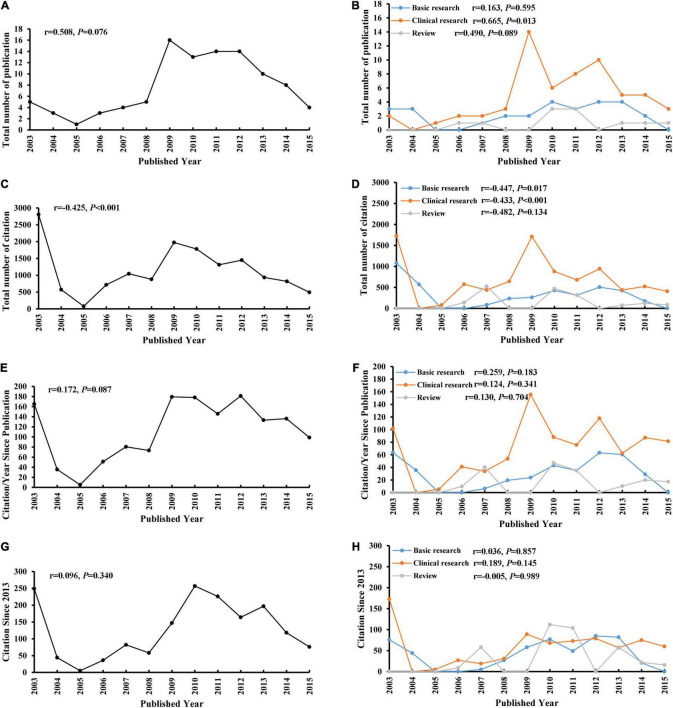
Publication and citation according to published year. **(A)** total number of publication; **(B)** total number of publication per type of article; **(C)** total number of citation; **(D)** total number of citation per type of article; **(E)** citation/year since publication; **(F)** citation/year since publication per type of article; **(G)** citation since 2013; and **(H)** citation since 2013 per type of article.

The highest number of articles was 16 published in 2009. The highest total number of citations was 2,806 published in 2003. The highest number of citations/year since publication was 179.27 published in 2009. The highest number of citations since 2013 was 249 published in 2003 (Figure 1). The number of clinical research increased with an increase overtime (*r* = 0.665, *p* = 0.013). The total number of citations decreased for basic research (*r* = –0.447, *p* = 0.017) and clinical research (*r* = –0.433, *p* < 0.001) overtime. No significant correlation was found for the citations/year since publication and citations since 2013 overtime (all *p* > 0.05).

### Distribution of Journal and Country

**FIGURE 2:**
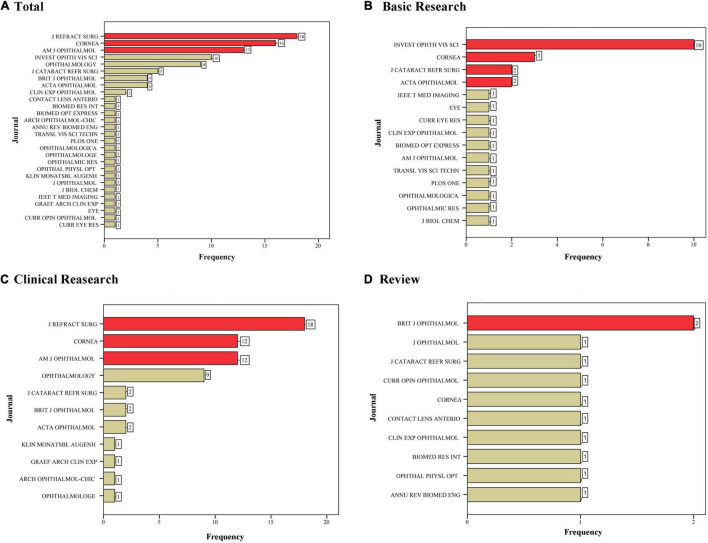
Journals of the 100 most-cited articles were published. **(A)** total; **(B)** basic research; **(C)** clinical research; and **(D)** review.

**FIGURE 3:**
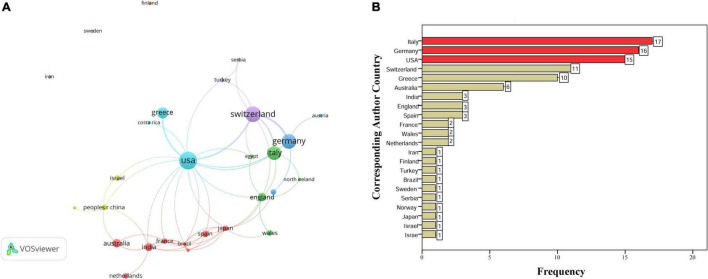
Country distributions of the 100 most-cited papers. **(A)** The collaboration networks of all authors country, and **(B)** corresponding author country.

The 100 most-cited articles were published in 28 journals, with the *J REFRACT SURG* publishing the highest number of articles (*N* = 18, Figure 2). The total number of citations, citations/year since publication, and citations since 2013 for all articles published in the journal were 1990, 207.06, and 110, respectively. The highest number of basic research, clinical research, and reviews were published in the *INVEST OPHTH VIS SCI* (*N* = 10), *J REFRACT SURG* (*N* = 18), and *BRIT J OPHTHALMOL* (*N* = 2), respectively. The 100 most-cited articles were from 28 countries, and United States has the highest number of articles (*n* = 30) and total link strength (Figure 3A and Supplementary Table 2). The corresponding authors of the 100 most-cited articles were from 22 countries, predominantly from Italy (*N* = 17), Germany (*N* = 16), and the United States (*N* = 15, Figure 3B).

### Analysis of Authors

The results show the collaboration networks and co-authorship map of 310 authors in the list of 100 most-cited articles (Supplementary Figure 1). Among the 71 authors with multiple authorships (*N* ≥ 2), Spoerl Eberhard has the highest number of citations and total link strength with 15 articles, followed by Seiler Theo that has 13 documents (Table 2). Furthermore, Wollensak Gregor had highest number of articles with first authorships or corresponding authorships (10 papers, Supplementary Figure 2).

**TABLE 2:** The top 10 authors of collaboration networks according to the number of documents.

ID	Author	Label x	Label y	Documents	Links	Total link strength	Citations	Avg. citations
1	Spoerl Eberhard	0.2072	–0.0134	15	300	1,613	4,477	298.47
2	Seiler Theo	0.1874	0.0813	13	301	1,720	4,689	360.69
3	Wollensak Gregor	0.0841	0.1186	11	289	1,316	3,649	331.73
4	Hafezi Farhad	0.5579	–0.0277	10	191	526	1,244	124.4
5	Vinciguerra Paolo	–0.3051	0.0999	8	195	502	924	115.5
6	Baiocchi Stefano	–0.4929	–0.0757	6	199	632	1,368	228
7	Caporossi Aldo	–0.5939	–0.1982	6	199	632	1,368	228
8	Kymionis George d	–0.5527	0.6799	6	91	245	531	88.5
9	Trazza Silvia	–0.2524	0.1966	6	171	408	756	126
10	Albe, Elena	–0.0264	0.181	5	164	359	664	132.8

### Results of the Keyword Co-occurrence

**FIGURE 4:**
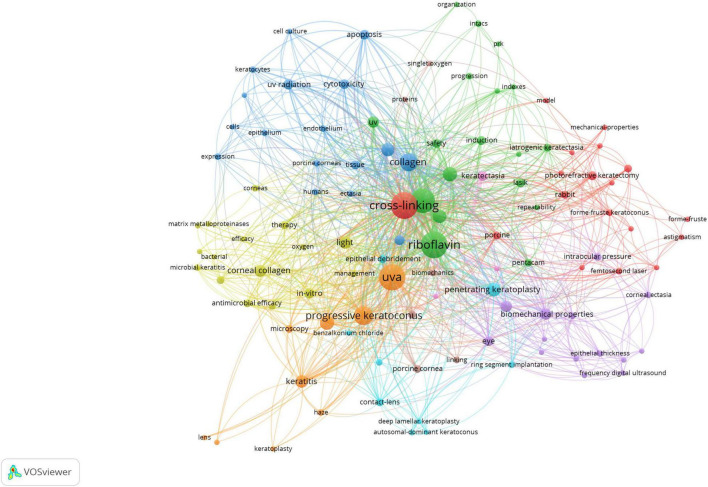
The co-occurrence network of keywords. The node represents the keyword, and its size is associated with the occurrence counts of the keyword. The link between two nodes means the co-occurrence of the two keywords.

The co-occurrence network of keywords that occur more than one time is showed in Figure 4. The top 10 keywords of the cooperation network were presented in Supplementary Table 3, with the most commonly used keywords were exhibited “cross-linking (*N* = 45),” “riboflavin (*N* = 44),” and “UVA (*N* = 42).” There were 110 keywords with multiple occurrences (*N* ≥ 2) of the total 322 keywords in the 100 most-cited articles, which could be classified into nine clusters (Supplementary Table 4).

## Discussion

A bibliometric analysis of top 100-cited articles could help researchers better know the key information of a specific field (22). The current bibliometric analysis of the 100 most-cited CXL articles showed that the highest number of publications occurred in 2009, and the highest total number of citations was published in 2003, and the *J REFRACT SURG* publishing the highest number of articles. In addition, “cross-linking,” “riboflavin,” and “UVA” were the most commonly used keywords, which provide guidance in evaluating the CXL researches over the past seven decades.

The CXL uses riboflavin and UVA irradiation as activators to strengthen and improve the biomechanical properties of corneas (23). The most-cited articles, which had 1,619 citations, were a plot study, reporting the effect of CXL utilization in 22 progressive keratoconus patients (2). The study was proposed early and was the basis of CXL research that provides guidance for later studies.

Among the top 100-cited articles, the majority was clinical research, and the number of clinical research was increased over time. It could be explained that the clinical research was original study that has scientific design to guarantee the reliability of the conclusion. In addition, with the improvement of surgery techniques, the number of patients receiving CXL is increasing, so the clinical researches in evaluating the effect of the surgery have also increased over time (24, 25). The citations of a paper, an important indicator reflecting the scientific merit, were reported to be affected by the research type (26). In the current study, the citations since 2013 of clinical research were lower than basic research and the reviews, which might be attributed to that review summarizes previously published data and literatures, and basic research mainly focused on explaining the mechanism of CXL surgery (25, 27). Furthermore, the total number of citations of basic research and clinical research were decreased over time. The phenomenon might be explained by that there exists a time interval from publication to citation, and papers conducted in earlier years were likely to be cited (15).

Among the top 100 most-cited articles, 18% were published in the *J REFRACT SURG*, which is ranked second in the total 2061 publications in the former study (17). The journal has been a monthly peer-reviewed forum for more than 30 years, and the 2019 Impact Factor was 2.711. In addition, *CORNEA* and *AM J OPHTHALMOL* also published the most articles in the top 100 publications of CXL researches. It has been reported that the scientific knowledge was transmitted to the members of a profession through publications of the scientific literature (28). The access method, annual number of articles, and Impact Factor could affect the journal in which the most-cited paper is published (29, 30).

As an important aspect of the article, the corresponding author holds a special position on the published work (31). The country analysis of corresponding authors indicated that Italy was responsible for the majority of the top 100-cited articles, followed by Germany and United States. The United States produced the highest number of articles and total link strength on the current list, which was consistent with the previous bibliometric analyses of ophthalmological researches (15, 20, 32). The previous study found that the top three countries in terms of the number of published articles in 2061 were the United States, China, and Germany, reflecting the number of articles, while current results tend to reflect the country distribution of high-quality articles (17). Furthermore, two authors (Vinciguerra Paolo and Mazzotta Cosimo) from Italy have contributed significantly to the top 100-cited articles. Vinciguerra et al. (33) firstly evaluated the preoperative and postoperative refractive, topographic, and tomographic outcomes in eyes with progressive keratoconus, and then published several researches in exploring the effect of CXL surgery in later years (34–37). Mazzotta et al. (38, 39) evaluated the morphological and functional correlations of Italy keratoconus during the 2008–2012. Wollensak Gregor had the most corresponding author articles, similar to the former results, which was responsible for the majority of highly cited papers from Germany (2, 17, 23, 40, 41). In addition, there exists a co-authorship operative network with Spoerl Eberhard, Seiler Theo, and Wollensak Gregor as the main nodes. It is necessary to increase the cooperation of different countries and authors to further promote the development of CXL research in the future study.

It has been reported that keywords are important parameters to determine the research topic and help researcher’s search-relevant publications (42). In the 100 most-cited researches on CXL research, nine keywords clusters identified the primary groups of topics in the studies. These clusters mainly focused on “cross-linking (cluster 1, red-colored),” “riboflavin (cluster 2, green-colored),” “collagen (cluster 3, blue-colored),” “light (cluster 4, yellow-colored),” “biomechanical properties (cluster 5, purple-colored),” “penetrating keratoplasty (cluster 6, light blue-colored),” “UVA (cluster 7, orange-colored),” “oxygen (cluster 8, light blue-colored),” and “keratectasia (cluster 9, pink-colored).” The keyword co-occurrence analysis can reveal the internal structure of the related literature and the frontier discipline (20). It has been reported that riboflavin, UVA radiation, and oxygen are the three critical elements required for effective CXL to occur (6). The current results indicated that the mechanisms, laboratory studies, and follow-up effect of the surgery were the hot spots of 100 most-cited CXL researches (2, 23, 43). In addition, the surgery methodology gradually diversified with the development of technology. Researches evaluating the effect of epithelium-on vs. epithelium-off techniques, and standard vs. accelerated CXL techniques were increasing rapidly (44, 45). It is useful to consider the most frequently used keywords identified in CXL research when planning future research.

To our knowledge, this is the first bibliometric study to identify the most cited papers in corneal CXL research. However, some limitations should be noted. First, the current study used the WOS database, one of the most popular resources for researchers interested in the field of citation analysis, and other databases might have a different hierarchy for papers on CXL. Although the results could not be directly generalized to other databases, it provides a reference in reflecting the important articles to some extent. Second, the bibliometric analysis quantifies the number of citations that were influenced by published time and journal, and should be noted when used in clinical application. Last, the language affected the citation of the article, which was in English in the current analysis, and articles in other languages might also have high-quality articles were not included in the analysis. Thus, a more comprehensive review of several indexing databases and extensive studies are needed in the future.

## Conclusion

In conclusion, the study present major advances and changes in research regarding CXL research, and the results could serve as a guide enabling clinicians to understand CXL research over past decades better.

## Data Availability Statement

The original contributions presented in the study are included in the article/Supplementary Material, further inquiries can be directed to the corresponding authors.

## Author Contributions

SR and CP conceived and designed the study. KY and LX analyzed the data and took responsibility for the integrity and accuracy of the information. LX, MZ, SW, QF, YG, QW, and KY contributed to the reagents, materials, and analysis tools. KY, LX, CP, and SR drafted the manuscript. KY, YW, DZ, CP, and SR revised the manuscript. All authors have approved the final manuscript.

## Conflict of Interest

The authors declare that the research was conducted in the absence of any commercial or financial relationships that could be construed as a potential conflict of interest.

## Publisher’s Note

All claims expressed in this article are solely those of the authors and do not necessarily represent those of their affiliated organizations, or those of the publisher, the editors and the reviewers. Any product that may be evaluated in this article, or claim that may be made by its manufacturer, is not guaranteed or endorsed by the publisher.

## Footnotes

1
www.vosviewer.com
