# Supplementary material for: A Bibliometric Analysis of 100 Most-Cited Articles on Corneal Cross-Linking
Source: Front Med (Lausanne). 2022 Jun 1;9:904077. doi: 10.3389/fmed.2022.904077 (PMC9199002; doi:10.3389/fmed.2022.904077)
Supplement: Supplementary file 1 [file Data_Sheet_1.PDF]

Supplementary Material

Supplementary Table 1 The 100 articles with the highest citation in corneal CXL.

Rank	Title	PMID	Total Citation	Citation/Year Since Publication	Citation Since 2013
1	Riboflavin/ultraviolet-A-induced collagen crosslinking for the treatment of keratoconus	12719068	1619	95.23529	167
2	Stress-strain measurements of human and porcine corneas after riboflavin-ultraviolet-A-induced cross-linking	14522301	661	38.88235	53
3	Safety of UVA-riboflavin cross-linking of the cornea	17457183	524	40.30769	58
4	Long-term Results of Riboflavin Ultraviolet A Corneal Collagen Cross-linking for Keratoconus in Italy: The Siena Eye Cross Study	20138607	428	42.8	39
5	Parasurgical therapy for keratoconus by rib oflavin-ultraviolet type A rays induced cross-linking of corneal collagen - Preliminary refractive results in an Italian study	16765803	311	22.21429	14
6	Keratoconus: A review	20537579	302	30.2	72
7	Refractive, Topographic, Tomographic, and Aberrometric Analysis of Keratoconic Eyes Undergoing Corneal Cross-Linking	19167087	285	25.90909	23
8	Keratocyte apoptosis after corneal collagen cross-linking using riboflavin/UVA treatment	14701957	274	17.125	13
9	Randomized controlled trial of corneal collagen cross-linking in progressive keratoconus: Preliminary results	18811118	273	22.75	4
10	Corneal cross-linking-induced stromal demarcation line	17133053	264	18.85714	13
11	Treatment of progressive keratoconus by riboflavin-UVA-induced cross-linking of corneal collagen - Ultrastructural analysis by Heidelberg Retinal Tomograph in vivo confocal microscopy in humans	17457184	262	20.15385	15
12	Endothelial cell damage after riboflavin-ultraviolet-A treatment in the rabbit	14522302	252	14.82353	15
13	Global Consensus on Keratoconus and Ectatic Diseases	25738235	234	46.8	38
14	A Randomized, Controlled Trial of Corneal Collagen Cross-Linking in Progressive Keratoconus Three-Year Results	24393351	204	34	30
15	Corneal healing after riboflavin ultraviolet-A collagen cross-linking determined by confocal laser scanning microscopy in vivo: Early and late modifications	18672225	187	15.58333	16
16	Ultraviolet A/riboflavin corneal cross-linking for infectious keratitis associated with corneal melts	18520510	184	15.33333	11
17	Comparison of Sequential vs Same-day Simultaneous Collagen Cross-linking and Topography-guided PRK for Treatment of Keratoconus	19772257	181	16.45455	11
18	Effect of inferior-segment Intacs with and without C3-R on keratoconus	17189797	176	13.53846	4
19	Mechanisms of Corneal Tissue Cross-linking in Response to Treatment with Topical Riboflavin and Long-Wavelength Ultraviolet Radiation (UVA)	19643975	175	17.5	27
20	Brillouin Optical Microscopy for Corneal Biomechanics	22159012	171	21.375	33
21	Keratocyte cytotoxicity of riboflavin/UVA-treatment in vitro	14739922	170	10.625	17
22	Antimicrobial efficacy of riboflavin/UVA combination (365 nm) in vitro for bacterial and fungal isolates: a potential new treatment for infectious keratitis	18408193	165	13.75	16
23	Corneal endothelial cytotoxicity of riboflavin/UVA treatment in vitro	14688422	165	9.705882	8
24	Photochemical Kinetics of Corneal Cross-Linking with Riboflavin	22427580	147	18.375	23
25	The Efficacy of Corneal Cross-Linking Shows a Sudden Decrease with Very High Intensity UV Light and Short Treatment Time	23299484	144	20.57143	21
26	Equivalence of Biomechanical Changes Induced by Rapid and Standard Corneal Cross-linking, Using Riboflavin and Ultraviolet Radiation	22025568	141	15.66667	9

27	High-Resolution Quantitative Imaging of Cornea Elasticity Using Supersonic Shear Imaging	19423431	140	12.72727	42
28	Transepithelial Corneal Collagen Cross-linking in Keratoconus	20166621	138	13.8	11
29	Post-laser in-situ keratomileusis ectasia: current understanding and future directions	16900036	138	9.857143	9
30	Permanent Corneal Haze After Riboflavin-UVA-induced Cross-linking in Keratoconus	19772259	134	12.18182	4
31	Contralateral Eye Study of Corneal Collagen Cross-linking With Riboflavin and UVA Irradiation in Patients With Keratoconus	19431928	134	12.18182	6
32	Use of Anterior Segment Optical Coherence Tomography to Study Corneal Changes After Collagen Cross-linking	19781685	133	12.09091	10
33	Corneal Collagen Cross-linking with Riboflavin and Ultraviolet A Irradiation for Keratoconus Long-term Results	23583165	129	18.42857	20
34	Intraoperative and Postoperative Effects of Corneal Collagen Cross-linking on Progressive Keratoconus	19822840	125	11.36364	2
35	Thermomechanical behavior of collagen-cross-linked porcine cornea	15004504	125	7.8125	14
36	Long-term biomechanical properties of rabbit cornea after photodynamic collagen crosslinking	18547280	122	11.09091	16
37	Biomechanics of corneal ectasia and biomechanical treatments	24774009	120	20	22
38	Management of keratoconus: current scenario	20693553	118	13.11111	28
39	Corneal Biomechanics and Biomaterials	21568714	117	13	69
40	Simultaneous Topography-guided PRK Followed by Corneal Collagen Cross-linking for Keratoconus	19772256	115	10.45455	7
41	UVA-riboflavin photochemical therapy of bacterial keratitis: a pilot study	21874347	113	14.125	16
42	Corneal Biomechanical Changes after Collagen Cross-Linking from Porcine Eye Inflation Experiments	20335615	113	11.3	30
43	Progression of Keratoconus and Efficacy of Pediatric Corneal Collagen Cross-linking in Children and Adolescents	23347367	112	14	3
44	Treatment of keratoconus by collagen cross linking	12557025	109	6.411765	6
45	Two-Year Corneal Cross-Linking Results in Patients Younger Than 18 Years With Documented Progressive Keratoconus	22633357	108	13.5	12
46	The Biomechanical Effect of Corneal Collagen Cross-Linking (CXL) With Riboflavin and UV-A is Oxygen Dependent	24349884	106	15.14286	2
47	Management of Corneal Ectasia After LASIK With Combined, Same-day, Topography-guided Partial Transepithelial PRK and Collagen Cross-linking: The Athens Protocol	21117539	105	11.66667	11
48	Riboflavin-UVA-Induced Corneal Collagen Cross-linking in Pediatric Patients	22420024	103	12.875	8
49	Corneal Biomechanical Properties at Different Corneal Cross-Linking (CXL) Irradiances	24677109	102	17	7
50	A randomised, prospective study to investigate the efficacy of riboflavin/ultraviolet A (370 nm) corneal collagen cross-linkage to halt the progression of keratoconus	21349938	102	11.33333	6
51	Proteases, proteolysis and inflammatory molecules in the tears of people with keratoconus	22413749	100	12.5	11
52	Effects of Ultraviolet-A and Riboflavin on the Interaction of Collagen and Proteoglycans during Corneal Cross-linking	21335557	99	11	15
53	Corneal Collagen Cross-linking With Riboflavin and Ultraviolet-A Irradiation in Patients With Thin Corneas	21861976	97	12.125	8
54	Riboflavin and Ultraviolet Light A Therapy as an Adjuvant Treatment for Medically Refractive Acanthamoeba Keratitis Report of 3 Cases	20884060	97	10.77778	12
55	Scheimpflug Imaging of Corneas After Collagen Cross-Linking	19421048	97	8.818182	2
56	Collagen Cross-Linking with Photoactivated Riboflavin (PACK-CXL) for the Treatment of Advanced Infectious Keratitis with Corneal Melting	24576886	96	16	15
57	Corneal Confocal Microscopy Following Conventional, Transepithelial, and Accelerated Corneal Collagen Cross-linking Procedures for Keratoconus	23347370	96	12	4

58	The Effect of Riboflavin/UVA Collagen Cross-linking Therapy on the Structure and Hydrodynamic Behaviour of the Ungulate and Rabbit Corneal Stroma	23349690	95	13.57143	25
59	Polymicrobial Keratitis After a Collagen Cross-Linking Procedure With Postoperative Use of a Contact Lens: A Case Report	19411973	93	8.454545	3
60	Corneal Cross-Linking as a Treatment for Keratoconus Four-Year Morphologic and Clinical Outcomes with Respect to Patient Age	23290750	92	13.14286	7
61	Collagen cross-linking: a new treatment paradigm in corneal disease - a review	20398104	92	9.2	25
62	Clinical and Corneal Biomechanical Changes After Collagen Cross-Linking With Riboflavin and UV Irradiation in Patients With Progressive Keratoconus: Results After 2 Years of Follow-up	22378112	90	11.25	13
63	One-Year Follow-up of Corneal Confocal Microscopy After Corneal Cross-Linking in Patients With Post Laser In Situ Keratosmileusis Ectasia and Keratoconus	19200532	90	8.181818	2
64	Current Protocols of Corneal Collagen Cross-Linking: Visual, Refractive, and Tomographic Outcomes	26008626	89	17.8	11
65	Dynamic OCT measurement of corneal deformation by an air puff in normal and cross-linked corneas	22435096	88	11	18
66	Collagen Cross-linking in Early Keratoconus With Riboflavin in a Femtosecond Laser-created Pocket: Initial Clinical Results	19731884	88	8	6
67	The Genetic and Environmental Factors for Keratoconus	26075261	86	17.2	16
68	Intraoperative Pachymetric Measurements during Corneal Collagen Cross-Linking with Riboflavin and Ultraviolet A Irradiation	19850346	85	7.727273	3
69	Transepithelial Versus Epithelium-off Corneal Cross-linking for the Treatment of Progressive Keratoconus: A Randomized Controlled Trial	25703475	84	16.8	11
70	Detection of Biomechanical Changes After Corneal Cross-linking Using Ocular Response Analyzer Software	21243976	84	9.333333	8
71	Stability of Simultaneous Topography-guided Photorefractive Keratectomy and Riboflavin/UVA Cross-linking for Progressive Keratoconus: Case Reports	20954679	84	8.4	7
72	Topography-guided Transepithelial Surface Ablation Followed by Corneal Collagen Crosslinking Performed in a Single Combined Procedure for the Treatment of Keratoconus and Pellucid Marginal Degeneration	20163079	84	8.4	1
73	Transepithelial corneal collagen cross-linking by iontophoresis of riboflavin	23848196	83	13.83333	19
74	Wound healing in the rabbit cornea after corneal collagen cross-linking with riboflavin and UVA	17525659	83	6.384615	5
75	Complications of Corneal Collagen Cross-Linking	22254130	81	9	7
76	Can We Measure Corneal Biomechanical Changes After Collagen Cross-Linking in Eyes With Keratoconus? A Pilot Study	19421050	81	7.363636	3
77	Collagen Cross-Linking Using Rose Bengal and Green Light to Increase Corneal Stiffness	23599326	78	11.14286	34
78	A new treatment of keratectasia after LASIK with riboflavin/UVA light cross-linking	15912463	78	5.2	5
79	Morphological and functional correlations in riboflavin UV A corneal collagen cross-linking for keratoconus	20456255	77	9.625	6
80	Collagen cross-linkage: a comprehensive review and directions for future research	19666925	77	7.7	15
81	Photoactivated Riboflavin Treatment of Infectious Keratitis Using Collagen Cross-linking Technology	23062001	76	9.5	6
82	Corneal Collagen Cross-Linking for Ectasia after LASIK and Photorefractive Keratectomy Long-Term Results	23582990	75	10.71429	10
83	Patient-Specific Computational Modeling of Keratoconus Progression and Differential Responses to Collagen Cross-linking	22039252	75	8.333333	25
84	Transient Corneal Thinning in Eyes Undergoing Corneal Cross-Linking	21726844	75	8.333333	11
85	Corneal Endothelial Damage After Collagen Cross-Linking Treatment	22001813	74	8.222222	3

86	Intra- and Postoperative Variation in Ocular Response Analyzer Parameters in Keratoconic Eyes After Corneal Cross-linking	20438025	74	7.4	3
87	Effects of riboflavin/UVA corneal cross-linking on keratocytes and collagen fibres in human cornea	20447101	74	7.4	8
88	Simultaneous Topography-Guided Photorefractive Keratectomy Followed by Corneal Collagen Cross-linking for Keratoconus	21794846	73	8.111111	2
89	Corneal Cross-linking: Intrastromal Riboflavin Concentration in Iontophoresis-Assisted Imbibition Versus Traditional and Transepithelial Techniques	24321474	72	12	14
90	Long-term follow-up of riboflavin/ultraviolet A (370 nm) corneal collagen cross-linking to halt the progression of keratoconus	23385632	72	10.28571	11
91	Corneal cross-linking - a review	23406488	72	10.28571	58
92	Corneal Collagen Cross-linking for Ectasia After Excimer Laser Refractive Surgery: 1-year Results	19772221	72	7.2	7
93	Transepithelial Iontophoresis Corneal Collagen Cross-linking for Progressive Keratoconus: Initial Clinical Outcomes	25375847	71	11.83333	3
94	Combined Transepithelial Phototherapeutic Keratectomy and Corneal Collagen Cross-Linking for Progressive Keratoconus	22683058	71	8.875	3
95	Corneal Cross-linking with Hypo-osmolar Riboflavin Solution in Thin Keratoconic Corneas	21529763	70	7.777778	20
96	Keratoconus Management: Long-Term Stability of Topography-Guided Normalization Combined With High-Fluence CXL Stabilization (The Athens Protocol)	24763473	69	11.5	8
97	Epithelium-Off Corneal Collagen Cross-linking Versus Transepithelial Cross-linking for Pediatric Keratoconus	23132450	69	9.857143	9
98	Refractive and Topographic Results of Transepithelial Cross-Linking Treatment in Eyes With Intacs	19574920	69	6.272727	7
99	Gel electrophoretic analysis of corneal collagen after photodynamic cross-linking treatment	18362667	69	5.75	11
100	Pharmacological Modification of the Epithelial Permeability by Benzalkonium Chloride in UVA/Riboflavin Corneal Collagen Cross-Linking	20673048	68	6.8	12

Supplementary Table 2 The top 10 countries of collaboration networks according to the number of documents

Id	Country	label x	label y	Documents	Links	Total link strength	Citations	Avg. citations
1	USA	-0.0348	0.0501	30	16	29	3825	127.5
2	Switzerland	0.451	0.4007	23	7	27	5781	251.3478
3	Germany	0.7212	0.1979	20	4	16	5037	251.85
4	Italy	0.6109	0.1081	18	4	9	2645	146.9444
5	Greece	-0.2293	0.4116	11	1	6	1058	96.1818
6	Australia	-0.5746	-0.5734	7	3	4	976	139.4286
7	England	0.5214	-0.2245	7	8	9	882	126
8	India	-0.3344	-0.6014	6	9	10	696	116
9	Spain	0.0926	-0.5013	4	7	7	737	184.25
10	France	-0.2129	-0.5548	3	7	7	470	156.6667

Supplementary Table 3The top 10 keywords of collaboration networks according to the number of occurrence

Id	Keyword	Label x	Label y	Occurrences	Links	Total link strength	Avg. citations
1	cross-linking	-0.1135	0.1388	45	94	292	169.2444
2	riboflavin	0.0214	-0.0397	44	87	277	114.5
3	uva	-0.1702	-0.1861	42	83	251	104.8571
4	keratoconus	-0.0306	0.1621	38	84	230	134.7895
5	progressive keratoconus	-0.3021	-0.3616	23	46	129	104.913
6	collagen	-0.0954	0.3384	20	56	129	186.9
7	in-situ keratomileusis	0.092	0.2849	13	52	98	258.3846
8	in-vivo	-0.4686	-0.3946	13	37	70	131.2308
9	riboflavin/uva	0.0456	0.0895	12	44	84	143.4167
10	penetrating keratoplasty	0.295	-0.2449	12	42	70	170.1667

Supplementary Table 4 The keywords clusters of collaboration networks

Cluster	Keyword	Label x	Label y	Links	Total link strength	Occurrences	Avg. citations
1	cross-linking	-0.1135	0.1388	94	292	45	169.24
1	photorefractive keratectomy	0.7446	0.2753	28	33	5	96.60
1	porcine	0.3076	0.0033	23	31	4	94.75
1	rabbit	0.618	0.1885	20	22	4	89.50
1	risk-factors	0.9075	0.3078	19	25	4	120.00
1	femtosecond laser	0.867	-0.1613	17	21	3	164.00
1	forme-fruste keratoconus	0.8228	0.1058	20	24	3	105.33
1	anterior lamellar keratoplasty	1.0367	-0.1432	10	13	2	176.00
1	applanation tonometry	0.7786	0.4121	13	15	2	99.00
1	astigmatism	1.1511	-0.0052	8	8	2	159.00
1	follow-up	0.6979	-0.1424	16	16	2	122.50
1	forme-fruste	1.1804	0.0763	3	3	2	99.50
1	mechanical-properties	0.8	0.4973	12	13	2	115.00
1	model	0.5347	0.6183	12	14	2	97.50
1	ocular response analyzer	0.8341	0.2426	15	16	2	100.50
1	refractive surgery	0.6513	0.3807	16	17	2	97.50
1	thickness	0.6018	0.2871	9	9	2	76.00
1	topography-guided prk	0.9197	0.0344	13	15	2	119.00
2	riboflavin	0.0214	-0.0397	87	277	44	114.50
2	keratoconus	-0.0306	0.1621	84	230	38	134.79
2	in-situ keratomileusis	0.092	0.2849	52	98	13	258.38
2	riboflavin/uva	0.0456	0.0895	44	84	12	143.42
2	uv	-0.2546	0.5213	30	52	8	166.88
2	induction	0.2384	0.4354	20	26	5	296.00
2	lasik	0.4173	0.2442	23	31	5	110.80
2	safety	0.0321	0.4236	19	31	5	92.00
2	iatrogenic keratectasia	0.5399	0.4044	22	32	4	90.75
2	pentacam	0.4514	-0.1227	20	29	4	133.50
2	intacs	0.2242	0.9692	8	8	3	154.67
2	progression	0.1817	0.7308	13	15	3	593.33
2	indexes	0.3843	0.6822	14	15	2	72.00
2	laser	0.3109	0.6625	14	15	2	72.00
2	organization	0.1251	1.0585	5	5	2	286.50
2	prk	0.3158	0.8782	7	7	2	76.50
2	repeatability	0.4916	0.132	11	13	2	90.00
2	topography	0.4094	0.3728	14	15	2	91.50
3	collagen	-0.0954	0.3384	56	129	20	186.90
3	cornea	-0.1908	0.3965	47	85	11	151.36
3	confocal microscopy	-0.135	-0.0186	25	35	7	165.29
3	uv radiation	-0.6432	0.6913	23	47	7	452.71
3	apoptosis	-0.298	0.9203	20	41	6	202.17
3	cytotoxicity	-0.3885	0.6961	28	48	6	168.17

3	tissue	-0.3367	0.3243	20	32	5	234.60
3	ectasia	-0.3591	0.1953	15	18	3	104.33
3	endothelium	-0.4848	0.4917	19	28	3	252.33
3	epithelium	-0.7512	0.4708	11	12	3	194.33
3	expression	-0.9654	0.3618	15	18	3	85.67
3	humans	-0.521	0.2015	6	9	3	203.33
3	keratocytes	-0.8063	0.7612	15	22	3	175.67
3	porcine corneas	-0.5135	0.3324	22	30	3	111.33
3	cell culture	-0.6384	0.9359	11	16	2	167.50
3	cells	-0.8997	0.4984	12	13	2	174.00
3	keratocyte apoptosis	-0.843	0.6435	10	11	2	167.50
4	light	-0.3847	-0.0291	34	58	10	104.20
4	corneal collagen	-0.7731	-0.1576	25	50	9	278.78
4	in-vitro	-0.5464	-0.2599	31	45	7	104.86
4	antimicrobial efficacy	-0.8303	-0.307	21	34	4	98.25
4	inactivation	-0.9543	-0.1996	17	21	4	130.50
4	therapy	-0.6607	0.0499	25	33	4	190.25
4	corneas	-0.7955	0.2179	11	14	3	128.33
4	infectious keratitis	-0.7155	-0.3215	18	27	3	105.67
4	management	-0.3438	-0.1718	24	25	3	157.67
4	microbial keratitis	-1.0602	-0.1426	20	22	3	124.67
4	bacterial	-0.9825	-0.0952	13	14	2	86.00
4	efficacy	-0.8488	-0.0129	17	17	2	99.00
4	matrix metalloproteinases	-1.0071	0.0558	11	12	2	98.00
4	oxygen	-0.5773	-0.0491	13	15	2	143.00
4	proteases	-1.0559	0.0481	11	12	2	98.00
4	resistance	-1.0492	-0.0708	10	11	2	96.50
5	biomechanical properties	0.5306	-0.3555	40	58	8	168.13
5	rabbit cornea	0.3502	-0.3205	36	58	8	97.25
5	eye	0.2695	-0.479	27	38	6	87.83
5	intraocular pressure	0.7677	-0.0949	22	25	4	116.50
5	aberrations	0.6543	-0.3639	19	22	3	155.67
5	corneal ectasia	0.9984	-0.2703	18	21	3	149.00
5	epithelial thickness	0.7784	-0.537	18	24	3	91.33
5	3-dimensional display	0.8889	-0.5668	13	16	2	74.50
5	custom phototherapeutic keratectomy	0.5261	-0.4713	15	16	2	205.00
5	frequency digital ultrasound	0.8847	-0.6247	13	16	2	74.50
5	hysteresis	0.7075	-0.6367	11	12	2	79.00
5	riboflavin concentration	0.9704	-0.5258	8	9	2	73.00
5	rotating scheimpflug camera	0.5121	-0.5317	15	16	2	205.00
5	topography analysis	0.6707	-0.5611	13	16	2	99.50
6	penetrating keratoplasty	0.295	-0.2449	42	70	12	170.17
6	contact-lens	-0.2257	-0.7579	26	33	4	149.50
6	epithelial debridement	-0.2134	-0.1065	24	31	4	93.25

6	ring segment implantation	0.3752	-0.5866	21	24	3	235.00
6	vivo confocal microscopy	-0.2286	-0.5762	24	30	3	154.00
6	autosomal-dominant keratoconus	-0.0144	-0.896	9	12	2	194.00
6	benzalkonium chloride	-0.3663	-0.4425	15	17	2	103.00
6	collaborative longitudinal evaluation	-0.0798	-0.8975	9	12	2	194.00
6	deep lamellar keratoplasty	-0.0468	-0.8452	9	12	2	194.00
7	uva	-0.1702	-0.1861	83	251	42	104.86
7	progressive keratoconus	-0.3021	-0.3616	46	129	23	104.91
7	in-vivo	-0.4686	-0.3946	37	70	13	131.23
7	keratitis	-0.5836	-0.6626	31	56	10	112.60
7	microscopy	-0.639	-0.4254	15	26	4	81.75
7	haze	-0.4942	-0.8046	11	13	3	75.00
7	keratoplasty	-0.7139	-0.9728	5	5	2	103.50
7	lens	-1.0277	-0.9204	6	7	2	159.00
7	scattering	-0.9695	-0.9421	7	7	2	133.00
8	behavior	-0.08	-0.36	14	25	4	91.25
8	porcine cornea	-0.0532	-0.6057	17	24	4	93.25
8	biomechanics	0.0152	-0.1681	19	25	3	121.33
8	linking	0.1253	-0.5649	13	15	3	105.00
8	proteins	-0.1122	0.6231	11	13	2	122.00
8	singlet oxygen	0.0042	0.7879	10	11	2	126.50
9	keratectasia	0.2439	0.2744	25	39	6	179.83
9	pachymetry	0.0313	-0.2747	10	13	2	87.00
9	ultrasound	0.3089	-0.1503	14	15	2	118.50

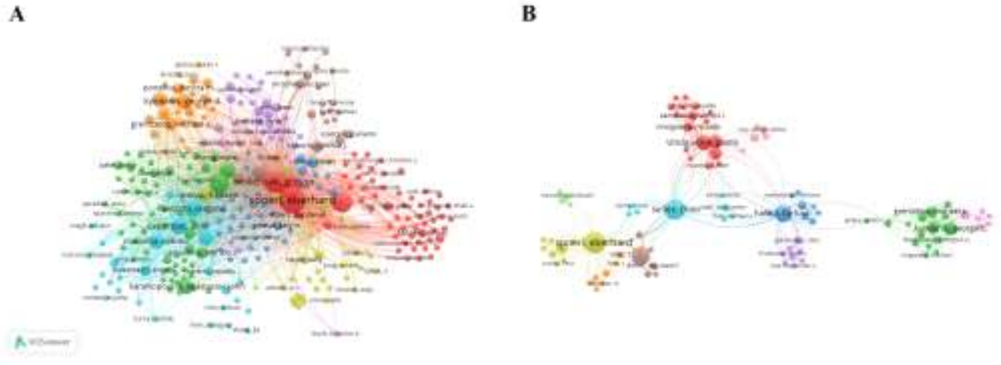

Supplementary Figure 1 The collaboration networks and co-authorship map of all authors. (A) collaboration networks map, (B) co-authorship map The nodes represent the authors. The size of the nodes is associated with the number of articles they published. The link between two nodes means that they had cooperation relationship.

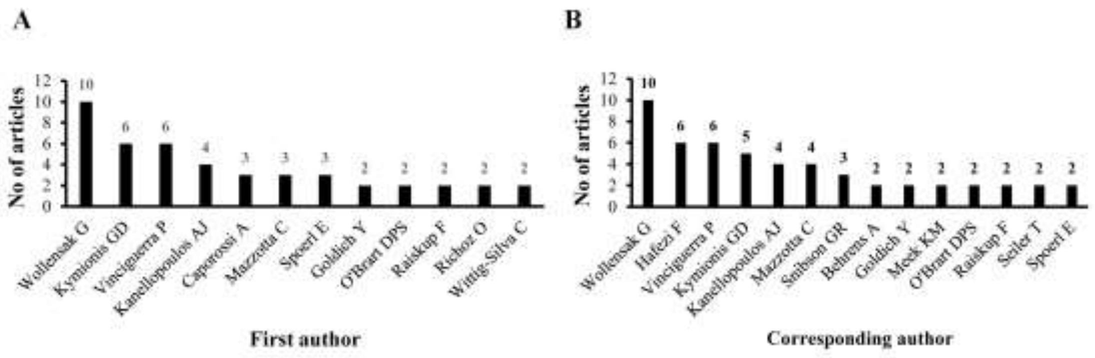

Supplementary Figure 2 First and corresponding authors with multiple articles. (A) First author; (B) corresponding author.
